# High-Quality Bioethanol and Vinegar Production from Saudi Arabia Dates: Characterization and Evaluation of Their Value and Antioxidant Efficiency

**DOI:** 10.3390/antiox11061155

**Published:** 2022-06-13

**Authors:** Zeineb Hamden, Yassine El-Ghoul, Fahad M. Alminderej, Sayed M. Saleh, Hatem Majdoub

**Affiliations:** 1 Laboratory of Interfaces and Advanced Materials, Faculty of Sciences of Monastir, University of Monastir, Monastir 5000, Tunisia; zeineb.hamden@yahoo.fr (Z.H.); hatem.majdoub@fsm.run.tn (H.M.); 2 Department of Chemistry, College of Science, Qassim University, Buraidah 51452, Saudi Arabia; e.saleh@qu.edu.sa; 3 Textile Engineering Laboratory, University of Monastir, Monastir 5000, Tunisia; 4 Chemistry Branch, Department of Science and Mathematics, Faculty of Petroleum and Mining Engineering, Suez University, Suez 43721, Egypt

**Keywords:** Saudi Khalas dates, bioethanol, vinegar, antioxidant activity, TPC evaluation, β-carotene assay, DPPH assessment, FRAP assay

## Abstract

Dates are very rich in various nutritious compounds, especially reducing sugars. Sugars ensure both anaerobic and aerobic fermentation, carried out respectively for the production of bioethanol and vinegar. Currently, the world production of dates is constantly increasing owing to the significant improvement in production conditions following the continuous scientific and technological development of this field. The Kingdom of Saudi Arabia is one of the most important world producers of dates, occupying the second place by producing 17% of the total world production. This is why it has become a national priority to find new ways to exploit and further valorize dates and palm waste in the development of new and sustainable products. The present study was designed to explore the possible study of a variety of date palm by-products in the production of bioethanol and vinegar via *Saccharomyces cerevisiae*. Different parameters of bioethanol and vinegar production, including pH, time, fermentation temperature, and yeast concentration, were studied and optimized. Chemical, physicochemical, purity behavior, and antioxidant performance were carried out via NMR, FTIR, and antioxidant activity essays (TPC, DPPH, FRAP, and β-carotene bleaching test) with the aim to evaluate the potential of the bioethanol and vinegar samples extracted from date palm by-products. Khalas date vinegar revealed significantly more phenolic content (5.81 mg GAE/mL) (*p* < 0.05) than the different kinds of vinegar tested (Deglet Nour and Black dates; 2.3 and 1.67 mg GAE/mL, respectively) and the commercial vinegar (1.12 mg GAE/mL). The Khalas date vinegar generally showed a higher carotenoid value and better antioxidant activity than the other vinegars extracted from other date varieties and commercially available vinegar. The results confirmed the high quality of the bioethanol and vinegar products, and the efficiency of the developed production processes.

## 1. Introduction

The date palm (*Phoenix dactylifera* L.) is one of the oldest plants cultivated in the world. Its cultivation had taken place 5000 years ago [1]. It grows in warm arid and half-arid areas of the earth. The date palm is the most abundant agricultural product in the world’s arid areas, especially in the region of Mideast, North Africa, and Maghreb. Due to the extension of its agricultural areas, its favorable climate, and its particular soil, the Kingdom of Saudi Arabia is one of the largest world producers of dates. It is ranked second in the world in date production, accounting for 17% of total world production. In accordance with data from the FAO (Food and Agriculture Organization) source, since 2014, date production in Saudi Arabia has increased by 14.8% year-on-year to reach 1,310,015 metric tons in 2019 [2]. This progress in national production scales is regrettably accompanied by a sharp increase in the loss of dates during harvesting, preservation, or during the conditioning process.

In line with this, a recent study showed that the total loss of dates in Saudi Arabia during the date marketing process was estimated at 7.8%. This attests that approximately 60 thousand tons of dates were lost, with a total value of 698 thousand SAR (1 US$ = 3.75 SAR (Saudi Arabia Riyal)) annually [2].

Moreover, the level of waste losses that occur along the marketing chain differs from one region to another and from one variety to another; for example, the amount of waste of “Khalas” variety in the Qassim region is over 10% while it does not exceed 5% in the Al-Ahsa region. Lost dates are not consumed by humans for several reasons such as the presence of a hard, defective, and poor quality texture, or their contamination by fungi, microorganisms or insects. These date by-products are usually rejected or, used for animal feed in some rare cases [3]. These abandoned date fruits are highly rich in biodegradable sugars which is a mixture of glucose and fructose of about 62.2% on dry mass [4]. Thus, their offal, rich in sugars, could be transformed by certain biotechnical processes in order to obtain various products such as; baker’s yeast [5], biofuels (hydrogen, butanol), alcohol, and vinegar.

Bioethanol or ethyl alcohol is a valuable green fuel which has a number of benefits when compared to conventional fuels. Cited as an example: it is biodegradable, very low in toxicity, and causes little environmental problem. In 2018, bioethanol produced globally reached 110 billion liters and it is estimated to reach about140 billion liters in 2022 with a compound annual growth rate (CAGR) of 7.6% in the light of economic and financial feasibility analysis of the process [6].

A large number of research reviews and articles describing different bioethanol production techniques and methods are present in the literature, research comprises enzymatic fermentation, enzymatic hydrolysis, simultaneous saccharification and fermentation procedure modeling and supply chain simulations [7,8]. The review of the bibliography also found that considerable research has been conducted on both sugar extraction and bioethanol generation from dates. The best key factors to improve ethanol efficiency are sugar concentration, pH of the fermentation medium, process time and temperature, yeast or enzyme stains, and shaker rpm. In order to increase ethanol production from date molasses, Alamri et al. studied the influence of a few parameters such as temperature, pH, and molasses concentration for ethanol production from date molasses. Ethanol production was improved using the yeasts *Hanseniaspora guilliermondii* (KKUY-0036) and *H. uvarum* (KKUY-0078) [9]. Chniti et al. tested the use of date syrup as a source of bioethanol produced by three dissimilar yeasts (*Saccharomyces cerevisiae*, *Zygosaccharomyces rouxii*, and *Candida pelliculosa*) [10]. Taken together, the medium’s sugar concentration and yeast strain have a significant effect on ethanol concentration in batch fermentation. In another study, Ghanim et al. manufactured bioethanol from DWs using hydrothermal extraction technology, aerobic and anaerobic fermentation processes, and distillation [11]. Bassam et al. examined the effect of two varieties of yeast: *S. cerevisiae* and *C. utilis* yeasts to ferment dates fruits. The results revealed that *S. cerevisiae* is able to metabolize rapidly date juice for ethanol production [12]. 

On the other hand, date vinegar is a natural product, used as a food additive, and has benefits for the treatment of many health problems. Numerous studies have reported the performance of fruit vinegar as a significant antioxidant agent that provides effective protection to functional organs against pathogenic flora. Vinegar from various fruits has proven anti-diabetic properties and the ability to decrease lipid concentration and blood pressure from carbohydrate food sources [13,14]. It is a natural product very rich in nutrients including vitamins and minerals such as magnesium, iron, silicon, calcium, phosphorus, and sulfur in addition to enzymes, essential acids, pectin, and some pigments [15].

Date vinegar has been used since the early years in the Middle East in cooking recipes as a food preservative and cleaning agent. Also, it has great application in medicine. In line with this perspective, Zeshan Ali and his colleagues found that date vinegar had advantageous effects on serum lipid profile parameters and inflammatory biomarkers in mildly hypercholesterolemic subjects [16]. The same research team indicated that date vinegar is mainly rich in phenolic compounds with potassium, folate, and organic acids. In this case, polyphenols can minimize the digestion and assimilation of fats, in the gastric and duodenal media [17]. In the same context, Wilcox et al. confirmed that polyphenols are involved in the control of critical intracellular enzymes that are vital for the production and secretion of Apo B lipoproteins [18]. All these data suggest the vigorous value of vinegar. 

Based on these considerations, the objective of the present research study focuses on the optimal conditions of bioethanol and vinegar production from by-product date palm cultivated in the Qassim region in Saudi Arabia country. The effects of different production conditions, including pH, time, fermentation temperature, and yeast concentration, were assessed and the different extracted products were characterized and their physicochemical and antioxidant properties were evaluated.

## 2. Materials and Methods

### 2.1. Raw Materials

The raw material is the Khalas” variety of Qassim dates (Buraidah, Saudi Arabia), which is considered to have a low market value. The dates used in this work are waste by-products (fruit with defects of texture, very moist fruits, fruits altered by microorganisms and insects). Palm dates are conserved at 4 °C until use.

### 2.2. Sugar Extraction

The liquid juice obtained during direct extraction goes through several steps starting with washing and pitting (kernel separation), then digestion with water (at ratio 1:3) and heating, which leads to the extraction of sugar and preparation of liquid juice. 

**Scheme 1:**
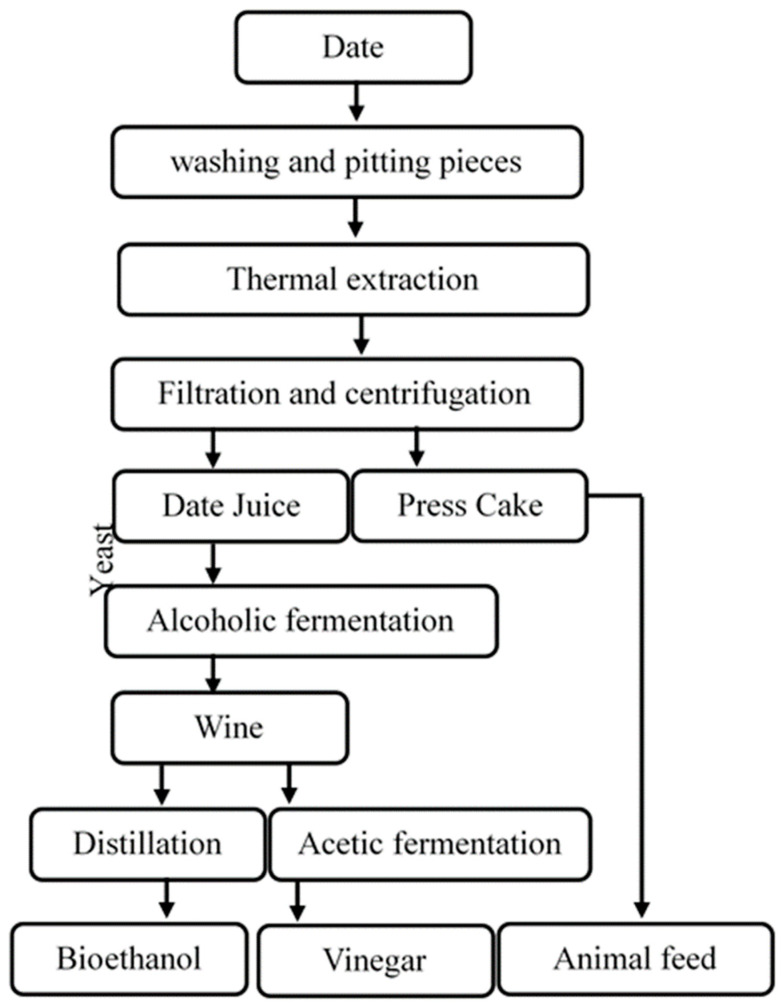
Different steps used for bioethanol and vinegar production.

Once the extraction time has elapsed, the whole is filtered and centrifuged to separate cellulose fibers then cooled and diluted to a concentration of 17% TSS which corresponds approximately to 162.2 g/L of sugar concentration. Finally, the prepared liquid juice was sterilized at 120 °C for 20 min. Otherwise, the centrifugation residue can be used as animal feed (Scheme 1).

### 2.3. Microorganisms and Growth Medium 

The microorganism saccharomyces cerevisiae yeast used in the alcoholic fermentation of date juice was purchased from “Rayen food industries” company (Tunis, Tunisia). It is a fresh active yeast, which is stored at 4 °C. The yeast *S. cerevisiae* is characterized by a good fermentation performance leading to rapid and high production of bioethanol from date juice [19].

For acetic fermentation, we use a mother of vinegar as acetic bacteria. These bacteria grow on the surface of the liquid during the wild fermentation of date juice exposed to the free air. The acetic bacteria transform alcohol into acetic acid using the oxygen in the air. They are added to date wine to produce vinegar. Five samples are prepared and denoted as B10, B15, B25, B50, and V15. The samples are cited in Table 1.

### 2.4. Preparation of the Fermentation Medium

Three liters of distilled water are added to 1 kg of washed dates, pitted, and cut into pieces of 0.5 cm each. The mixture is then heated at 85 °C for 45 min with continuous stirring. Afterward, it will be filtered through a sieve (0.5 mm) and centrifuged at 4000 rpm for 15 min. Total soluble solids (TSS) in the recovered dates juice were determined by a handheld refractometer for the HHTEC sugar brix refractometer model in (0–32% Brix) (HHTEC, Heidelberg, Germany) which represents approximately the total sugar concentration in g/L [20]. The initial pH of the juice is 5. It is therefore not necessary to carry out a correction of the pH of the juice since the optimum pH for alcoholic fermentation is between 4.5 and 5.5 [21]. 

### 2.5. Alcoholic Fermentation Test 

The Alcoholic fermentation has been carried out using the selected *S. cerevisiae* yeast. Bioethanol fermentation tests were conducted under anaerobic conditions in a round bottom flask (Florence Flask). The fermentation medium was then subjected to a fixed temperature of approximately 30 °C with continuous stirring using a magnetic stirrer. At constant time intervals, the wine was sampled for analysis.

The Bioethanol concentration in each sample was calculated based on the formula:

(1) Bioethanol concentration (g/L)=10×alcohol degree(°)×ρ

ρ  = density of ethanol = 0.789 g/L at a temperature of 20 °C

Centrifugation and distillation process 

After completing the alcoholic fermentation procedure, the fermentation broth was centrifuged to separate the solid biomass substrate from the liquid. Then, distillation was carried out at 78 °C to obtain the bioethanol samples.

### 2.6. Acetic Fermentation

Once the fermentable sugars present in the dates juice was transformed into bioethanol, the fermented jus was centrifuged at 4000 tours for 20 min. The next fermentation that takes place in the process is acetic fermentation, which consists of the oxidation of the alcohol into acetic acid providing it the characteristic vinegar taste. Appropriate vinegar bacteria called mother of vinegar was used for the acetification process. Mother of vinegar is perfectly adapted to date wine. 

### 2.7. Chemicals

Sodium hydroxide and absolute ethanol were pure chemicals from Sigma Aldrich (St. Louis, MO, USA). High purity quality solvents were purchased from Aldrich (St. Louis, MO, USA) and used without further purification.

### 2.8. Analysis

The different pH measurements of all prepared solutions: date juice, wine, and vinegar were determined using an Oakton PH 550 Benchtop pH meter Kit (Cole-parmer, Vernon Hills, IL, USA). 

A Gerber instruments AG digital thermometer (Gerber Instruments AG, Effretikon, Switzerland) was used for temperature control and measurements.

The degree of alcohol was determined via a Gerber instruments AG alcoholmeter (Gerber Instruments AG, Effretikon, Switzerland).

The total acidity was determined by potentiometric titration. A total of 10 mL of diluted vinegar samples (25-fold) was titrated with 0.1 M NaOH solution, according to [22].

Electrical conductivity was obtained using a DDS digital Lab conductometer (Thermo Fisher Scientific, Waltham, MA, USA). 

Infrared spectra were recorded with a Fourier transform infrared spectrophotometer BX FTIR system spectrometer (PerkinElmer, Waltham, MA, USA). Samples were analyzed in transmittance mode, and spectra were recorded in the 4500–400 cm^−1^ range. The system was equipped with a deuterated triglycine sulfate (DTGS) detector and UATR Diamond ATR (Single Reflection). Sixty-four scans were collected for each spectrum with 4 cm^−1^ resolution and 1 cm/s scan speed. Background spectra were collected prior to each measurement, according to Kadiroglu et al. [23]. 

The 1H NMR spectra of bioethanol and vinegar were obtained via an AVANCE-300 NMR spectrometer (Bruker Inc., Rheinstetten, Germany). Bioethanol samples were dissolved at 0.6 mL/mL in deuterated chloroform (CDCl3) (99.96%) before NMR analyses. For date vinegar, 0.1 mL of deuterated water (D2O) was added to 0.8 mL of vinegar sample. The 1H NMR (300 MHz) spectra were recorded at 40 °C.

Measurements were repeated two times for each sample. 

### 2.9. Antioxidant Activity of the Produced Date Vinegar

Four specific complementary assays (total phenolic content (TPC), DPPH, ferric reducing/antioxidant power (FRAP), and β-carotene bleaching) were carried out for the evaluation of the antioxidant potential of the prepared date vinegar.

All in vitro antioxidant assays were performed in triplicate and mean values were recorded.

#### 2.9.1. Total Phenolic Content Determination 

The determination of the total phenolic content (TPC) was conducted according to Folin–Ciocalteu’s method with some little modifications [24]. Gallic acid was used as a standard.

An aqueous solution was first prepared to contain a mixture of vinegar sample (50 μL), 3 mL ultrapure water, and 250 μL of the Folin-Ciocalteu’s reagent solution and well mixed. Then 750 μL of sodium carbonate (7%) was added. The solution is stirred continuously and then incubated for 10 min at room temperature before adding 950 μL of ultrapure water. After a 2 h incubation period, the absorbance was determined at 765 nm. The TPC was determined as gallic acid equivalents (mg GAE/mL). Linearity of the gallic acid calibration curve was obtained in the range of 50–1000 μg/mL (r = 0.99). The total phenolic contents are then deduced using the following formula:

(2) $$C=C1\times \frac{V}{\;m\;}$$

where C = total phenolic content in mg/g, in gallic acid equivalent, C1 = concentration of gallic acid settled from the calibration curve in mg/mL, V = volume of extract in ml, and m = the weight of vinegar extract in g.

#### 2.9.2. DPPH (1,1-Diphenyl-2-picrylhydrazyl) Radical Scavenging Activity

The DPPH free radical scavenging activity of dates’ vinegar was carried out as mentioned by Chen et al. with slight adjustments [25]. Indeed, 0.2 mL of sample was added to 3.8 mL of the ethanolic solution of DPPH radical (0.1 mM). After thorough stirring by vortex, the mixture was incubated in dark for 30 min. The absorbance of the sample was then measured at 517 nm against ethanol blank as a negative control. Ascorbic acid was used as a positive control. The measurements were expressed and calculated in micromoles of Trolox equivalents (TE) per ml of date vinegar (mg TE/mL).

#### 2.9.3. FRAP Assay

The Ferric Reducing/Antioxidant Power (FRAP) of the produced date vinegar was evaluated according to the method described by Szollosi et al. [26]. A sample of the produced date vinegar (50 µL) was mixed with 950 µL of a freshly prepared FRAP reagent (warmed at 37 °C) containing 25 mL of acetate buffer (0.3 µM pH 3.6), 2.5 mL of 10 µM TPTZ in 40 mM HCl, and 2.5 mL of 20 µM FeCl_3_·6H_2_O. After incubation for 10 min at 37 °C, the absorbance was measured at 593 nm. Ascorbic acid was used as a standard positive control. The FRAP value was expressed in AEAC (equivalent antioxidant capacity in ascorbic acid micromole per milliliter [µmol AEAC/mL]).

#### 2.9.4. Total Carotenoid Contents

The β-carotene bleaching test was carried out based on the method established by Sanusi and Adebiyi with slight modifications [27]. Concisely, the date vinegar sample (0.5 mL) was extracted with 8 mL of BHT (as a reference synthetic antioxidant) in ethanol (ethanol/butylated hydroxyl toluene 100:1, *v*/*w*). The extraction was repeated 3 times for the good separation and discharge of carotenoids. After being well mixed, it was kept in a water bath for 5 min at 85 °C. Then 0.5 mL of potassium hydroxide (80%) was added and the mixture was stirred before being placed back in the water bath at 85 °C. After 10 min, the preparation was cooled in an ice bath and 3 mL of demineralized water was added. After centrifugation, the yellow-colored supernatant was collected. Afterward, 12 mL of hexane solution was added to each centrifuge tube. The antioxidant activity was then determined according to the absorbance of the sample measured at 450 and 503 nm considering the hexane as the blank. The results were expressed as mg of total carotenoid content/100 mL. The following formula was applied to deduce the Total carotenoid content.

(3) $$Total\;carotene=4.642\times A_{450}\;-\;3.091\;\times \;A_{503}$$

### 2.10. Statistical Analysis

A one-way analysis of variance (ANOVA) was performed for the statistical analysis of the data. A Minitab software version 17 (Minitab Inc., State College, PA, USA) was used, and a level of *p* < 0.05 was considered to indicate the statistical significance of all experimental measurements. Analyzes were carried out in triplicate, and values were reported as mean ± standard deviations.

## 3. Results

### 3.1. Sugar Extraction and Evolution of Total Soluble Solids

**Figure 1:**
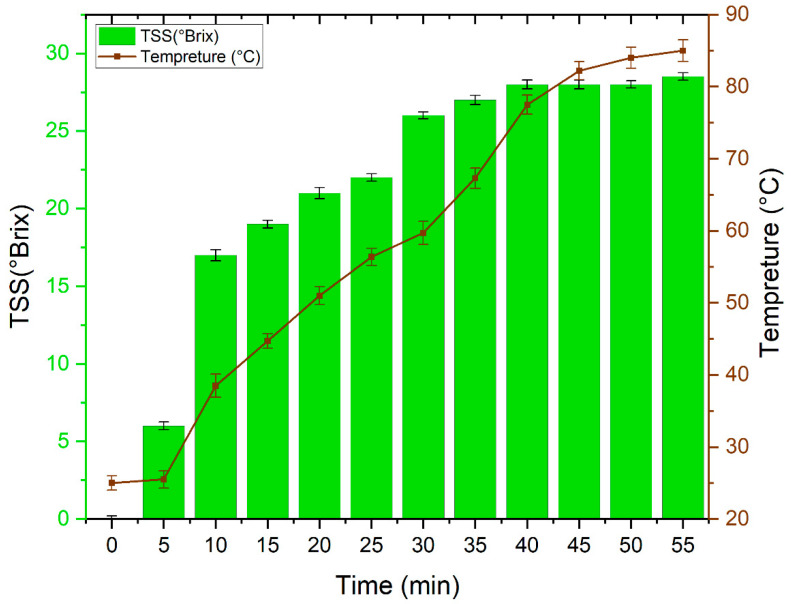
Evolution of Total Soluble Solids (TSS) with time during the direct sugar extraction at different temperatures.

Figure 1 displays the evolution of Total Soluble Solids (TSS) with time during the direct sugar extraction from the “Khalas” date variety at different temperatures (from room temperature to 85 °C). These results show that the sugar is extracted satisfactorily after 40 min and at a temperature of 77.5 °C. Furthermore, a careful analysis of the results obtained, allows us to distinguish the essential and significant role played by both temperature and time in the extraction of date juice. Indeed, the increase in temperature improves the permeability of solvents in fruit tissues and does so by disturbing the cellular structures, decreasing the viscosities, the surface tension, and the dielectric constant of the solvents and by increasing the solubility of the solutes [28,29,30]. This variation in temperature is accompanied by an increase in diffusion coefficients and mass transfer coefficients between solutes in fruits cells and solvents, which generates high extraction ability and productivities [31]. As well, high temperature affects the polar proprieties and character of water; it makes the polarity of water comparable to those of non-polar compounds. This character will increase the solubility of fewer polar compounds in water for extraction from different matrices [32].

### 3.2. Bioethanol Production

Table 2 illustrates the bioethanol concentration obtained throughout the alcoholic fermentation of the liquid date juice, after 72 h of fermentation at 30 °C, and by varying the concentration of the fresh *S. cerevisiae* yeast. It should be noted that the fermentation medium applied in this study did not undergo any enrichment, such as the addition of a source of nitrogen, proteins, mineral salts, or vitamins. 

The results presented in Table 2 show a significant increase in the alcohol concentration up to a yeast concentration of 25 g/L. Beyond this concentration, a considerable decrease in the concentration of the bioethanol produced was observed. A possible reason for this behavior is the important relationship between microorganisms and bioethanol concentration at the end of alcoholic fermentation. Indeed, a low yeast concentration leads to a deceleration of ethanol production, and the increase in yeast concentrations can result in competition between organisms which affect negatively the alcohol yield concentration [33]. The same results were reported by Ahmad et al. [34]. 

**Figure 2:**
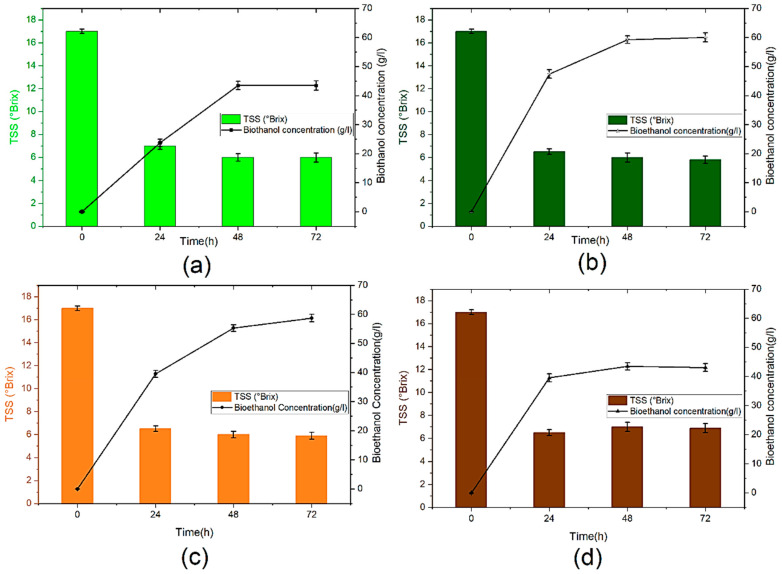
Bioethanol concentration and TTS (°Brix) content during anaerobic fermentation: (**a**): B10, (**b**): B15, (**c**): B25 and (**d**): B50.

Figure 2 displays the effect of yeast concentration on total soluble solids (TSS) consumption and bioethanol concentration. As shown in Figure 2 a continuous decrease in TSS during the fermentation process is accompanied by an important increase in the bioethanol concentration. However, the sugar is not completely consumed due to the termination of yeast growth affected by the aggregation and the accumulation of toxic substances into the wine, especially octane and decane in the fermentation medium [35]. This phenomenon may also be, justified by the fact that ethanol is recognized as an inhibitor of the growth of microbes; it has been proven that ethanol destroys the DNA of yeast cells and inactivates many enzymes [36].

Furthermore, after 48 h, we observed that the rate of bioethanol production and sugar consumption slowed down. This observed deceleration was due to the fact that the medium becomes more acidic after bioethanol production and carbon dioxide release as shown in the reaction below:

$$C_{6}H_{12}O_{6}\;(\mathrm{aq})\;\overset{Yeast}{\to }\;2C_{2}H_{5}\mathrm{OH}\;(\mathrm{aq})+2{\mathrm{CO}}_{2}\;(\mathrm{aq})\;$$

**Figure 3:**
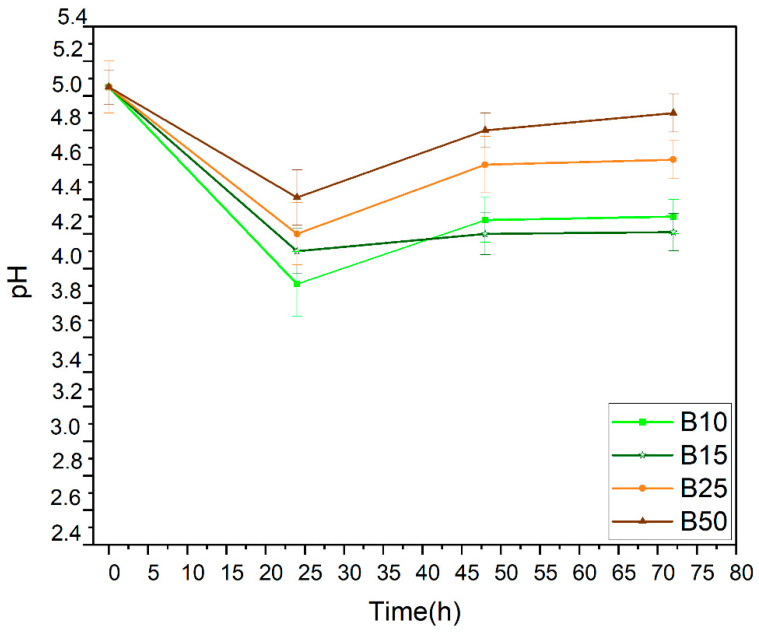
pH evolution during anaerobic fermentation.

This hypothesis can also be justified by the decrease in pH for all the samples tested during the first day of the fermentation process (Figure 3). This decrease is followed by a slight increase beyond 48 h. This experimental result can be explained by the formation of carbonic acid (weak acid) from CO_2_ and water according to the following reaction:

$${\mathrm{CO}}_{2}\;+\;H_{2}O\;\to \;H_{2}{\mathrm{CO}}_{3}$$

The raw bioethanol obtained by fermentation which is distilled and rectified at 78 °C, generated the highest ethanol concentration of approximately 93°; 94°; 94°, and 94° for the samples B10, B15, B2,5, and B50, respectively. Moreover, the maximum volume recovered from the bioethanol production is perceived for the sample “B15” which is equal to 272 mL/Kg. Nevertheless, the minimum volume of bioethanol production is illustrated for the sample “B50”. In this case, the recovered volume is 240 mL/ Kg. However, a previous study reported that the separation of fibers and their hydrolysis, lead to produce 20 mL of additional ethanol per 1 kg of dates, and consequently, the maximum yield can be increased from 272 to about 292 mL per Kg of DWs [37].

Table 3 summarizes the optimal results of the bioethanol production from dates obtained in this study and reports the different results presented in the literature. Table 3 also presents a comparative study of the various controlled conditions in the production of bioethanol and the concentration of optimal yield of bioethanol at the end of fermentation. These conditions include the initial pH, fermentation temperature, residence time, yeast stain, and stirring speed (shaker rpm). The comparison of the different data presented shows that the results obtained in our study are satisfactory and well comparable to those obtained by Chniti et al. and considerably more interesting than the other results found in the literature [10].

### 3.3. Infrared Spectroscopy Analysis 

**Figure 4:**
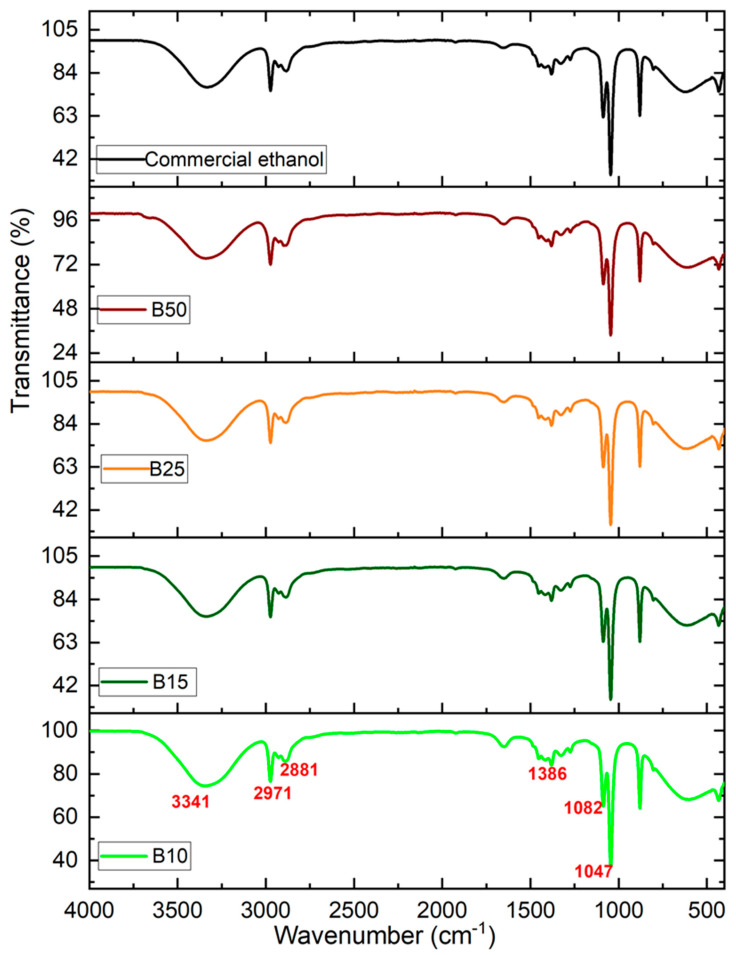
Infrared spectra of the commercial pure ethanol and the various produced bioethanol samples.

The FTIR spectrum of the bioethanol produced under the optimal production conditions is presented in Figure 4. The IR spectrum obtained is perfectly comparable to the spectrum of pure commercial ethanol.

The results for all analyzed samples show absorption bands typical of ethanol. Indeed, for all synthesized samples, a broad absorption band around 3341 cm^−1^, corresponds to the O-H stretching vibration of alcohols, thus the presence of the alcohol group is confirmed. The absorption bands at 2971 cm^−1^ and 2881 cm^−1^ were allotted to the C-H stretching vibration. The band at a wavenumber of approximately 1386 cm^−1^ is also detected. This absorbance is attributed to the C OH band of ethanol. In addition, the two characteristic bands that appeared around 1082 and 1047 cm^−1^ are associated with the stretching vibration of C-OH [39]. 

In order to obtain more structure information on the optimal bioethanol elaborated in the present study (B15), 1H NMR was studied. 

**Figure 5:**
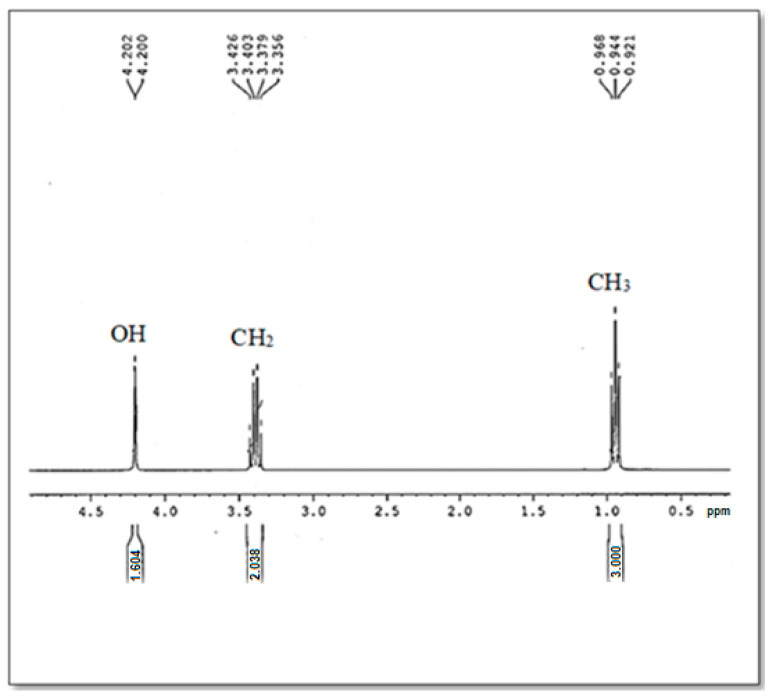
The 1H NMR Spectrum of the produced Bioethanol B15.

Referring to the 1H NMR chemical shifts analysis of the ethanol described in the bibliography [40,41], the chemical signals of bioethanol in the 1H NMR spectrum were identified (Figure 5). Indeed, the 1H NMR showed the presence of three chemical shifts centered at 1 ppm, 3.3 ppm, and 4.2 ppm which were assigned to CH_3_, CH_2_, and OH groups, respectively. So, the ethanol compound (C_2_H_5_OH) is confirmed.

### 3.4. Vinegar Production 

The aim of this phase is to produce high-quality date vinegar. Thus, a two-stage fermentation method (alcoholic and acetic) was applied for the production of vinegar from the Khalas variety of dates. In fact, after alcoholic fermentation, the wine was exploited to make vinegar using the mother of vinegar as the acetic acid bacteria. The physical and chemical analyses of date vinegar samples (pH, Brix, electrical conductivity, alcohol content, and the total acidity) are summarized in Table 4. As shown in Table 4, date vinegar is characterized by a relatively high electrical conductivity. In fact, the mean conductivity value of the elaborated sample was about 5650 µS/cm. This result is higher than those obtained in other previous works conducted by Siddeeg et al. and Akarca et al. with electrical values around 3100 and 4900 µS/cm respectively [42,43]. This difference may be related to the raw material used in vinegar production, fermentation time, and storage time after fermentation. Also, the pH values of vinegar samples were determined to be 3.6. A comparable result has been reported by Akarca et al. Additionally, in the present study, analysis of vinegar showed total acidity values of approximately 4%. Values that are confirmed by the north American legislation and set in more than 80% of the samples [44]. Moreover, no alcohol was detected in our vinegar samples, which is in good agreement with the 1H NMR analysis.

Therefore, all these data suggest that our product is characterized by high purity and has good similarity with previous research. 

### 3.5. IR Analysis 

**Figure 6:**
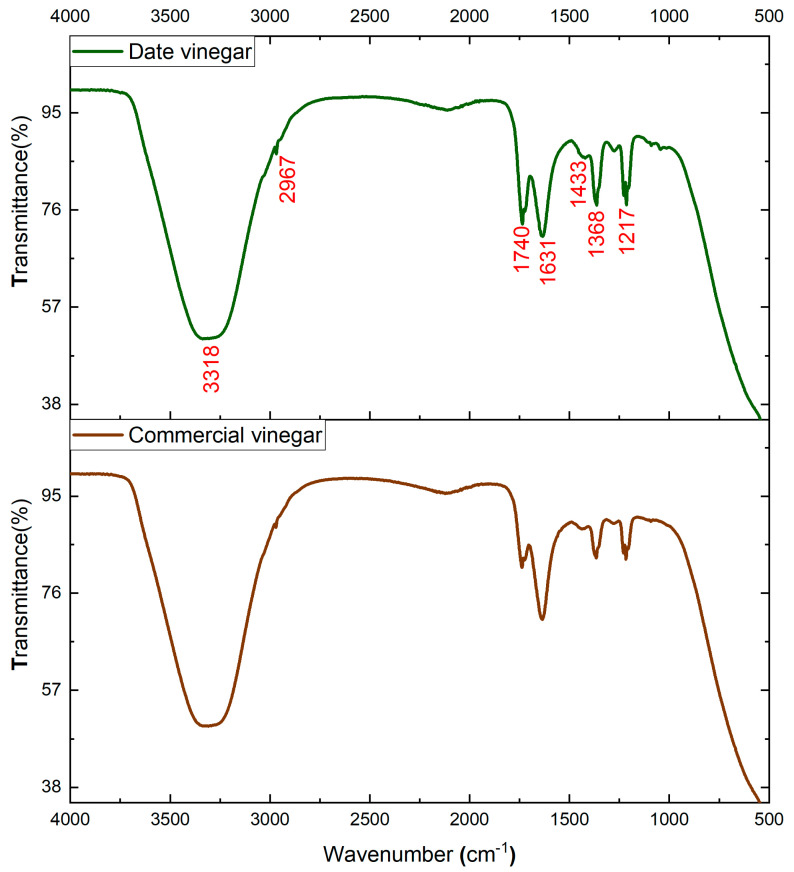
IR spectrum of date vinegar (V15) and commercial vinegar.

FTIR analysis is an easy, simple, and suitable analytical technique used for the identification and characterization of functional groups related to the major compounds that characterize date vinegar samples. Figure 6 shows the FTIR spectra of two analyzed vinegars. For a comparative purpose, a spectrum of commercial white vinegar is shown, as well as the spectrum of the produced date vinegar (V15). 

A careful analysis of Figure 6 reveals that the two analyzed samples show similar spectral fingerprints. Nevertheless, slight differences were found in the intensity of some bands. The main differences were detected usually for the bands at 1740, 1631, 1368, and 1217 cm^−1^. The large absorption band distinguished around 3318 cm^−1^ corresponds to strong water absorption with H-O-H stretching vibration. The small signals at 2967 cm^−1^ are assigned to C-H stretching vibrations of methyl- and methylene groups of carbohydrates or carboxylic acids [45]. 

Carboxylic acids are identified by C=O stretching associated with the bands at 1740 cm^−1^ and 1631 cm^−1^ [46]. The bands with a value of 1433 cm^−1^ are assigned to C–C stretching vibration in phenyl groups of aromatic compounds [47]. The signals at 1368 and 1217 cm^−1^ are assigned to various forms of C-O stretching vibrations of hydroxyflavonoids [46]. 

According to our results, date vinegar showed considerably higher intensities in these spectra bands than the commercial vinegar, suggesting the excellent quality of date vinegar and the discrimination among the commercial vinegar [48]. Furthermore, the major bands of date vinegar sample are assigned to acids and phenyl groups of aromatic compounds. These major bioactive ingredients in date vinegar are indeed correlated with the high antioxidant activity. Already, many research works have reported that the antioxidant activity of vinegar has a positive correlation with polyphenolic fraction. For instance, Verzelloni et al. [49] affirmed that the antioxidant capacity of red wines and traditional balsamic vinegar was highly correlated with their phenolic and flavonoid contents. Also, Vinegar contains abundant organic acids such as acetic acid, which are the main source of the strong aroma, vinegar flavor, and important indicators to evaluate the quality of vinegar [50].

### 3.6. H NMR Analysis

**Figure 7:**
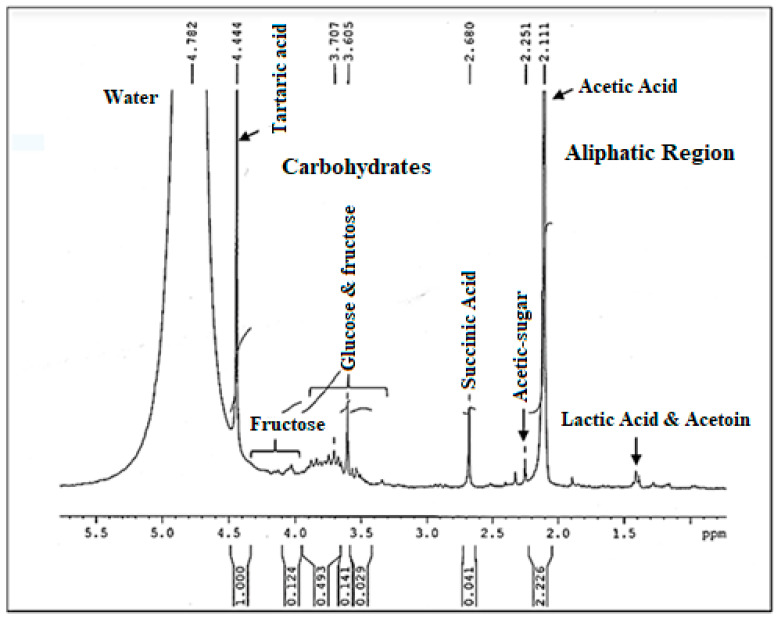
The 1H NMR Spectrum of the produced date vinegar V15.

NMR is the most powerful technique used to determine structural information of components in food complex systems. Figure 7 displays the 1H NMR spectrum, of the vinegar sample (V 15). First of all, it is important to point out that the 1H NMR spectrum of vinegar is characterized by a dominating water peak that is much greater than those of the components of interest, which causes some assignment problems. In fact, it can prevent the correct digitization of minor signals, hampering their observation and quantification. But, that does not prevent the profile spectrum from having a good resolution. Indeed, a detailed analysis of the 1H NMR spectrum of date vinegar (V15) showed a big presence of signals related to sugar compounds and organic acids. As shown in Figure 7 glucose and fructose signals are sited from 3.8 to 3.5 ppm. The peaks of fructose signals were located between 4 and 4.12 ppm. Furthermore, numerous organic acids are present in the aliphatic region of the 1H NMR spectrum of vinegar including lactic acid, succinic acid, and acetic acid emerged as the predominant ones. In addition, the signal of the acetic acid is well defined at 2.11 ppm and not overlapped with other peaks. Furthermore, tartaric acid is detected at 4.44 ppm. These last two acids lead in the vinegar spectrum. Signal assignments are listed in Table 5. Different mentioned groups are indicated according to the literature [51,52,53].

### 3.7. In Vitro Antioxidant Activity

#### 3.7.1. Total Phenolic Content Assay

Currently, plant materials rich in phenolics are used in the food industry thanks to their ability to decrease the oxidative degradation of lipids and maintain the quality and nutritional value of food [54]. Phenolic compounds in plants are also very important because their groups have scavenging abilities. They have largely proven their potential for health, mainly through their antioxidant activity. Their antioxidant action includes several mechanisms such as reduction of oxygen concentration, scavenging of initial radicals by transforming the initial products of oxidation into non-radical forms, and breaking the chains to avoid consistent cogitation of hydrogen from substrates [55]. The total phenolic contents of produced date vinegar, other date varieties, and commercial vinegar are shown in Table 6. Results revealed that our extracted date vinegar exhibited more phenolic content (5.81 mg GAE/mL) than the different tested kinds of vinegar, and the commercial vinegar (1.12 mg GAE/mL) (*p* < 0.05) as well as the Deglet Nour vinegar (2.3 mg GAE/mL) and black date vinegar (1.67 mg GAE/mL).

Compared to other studied date vinegar, the present khalas vinegar significantly showed higher total phenolic content. Indeed, it is reported that the TPC values of the Iranian variety Fardh were 641.17 mg GAE/L and 570.74 mg GAE/L for Honey variety [56], for Deglet Nour and Kentichi varieties were 230.9 and 209.42 mg of GAE per 100 g FW, respectively [57].

#### 3.7.2. Radical DPPH Scavenging Activity

It is well proven today that one of the causes and signs of cancer and other chronic diseases is the formation of free radicals in the body. These free radicals could be scavenged by the action of antioxidants in the diet and thereby the risk of disease would be reduced. in this perspective, the evaluation of the antiradical effect of the antioxidants present in the date vinegar would be essential and vitally important to evaluate its antioxidant potential. Table 6 shows the different DPPH values of commercial and produced date vinegars. Results revealed that the Khalas extracted date vinegar has a higher value of DPPH scavenging activity than the other vinegars (*p* < 0.05). Furthermore, compared to other reported date varieties, our produced vinegar exhibited a higher antiradical activity (2.01 mg TE/mL). Indeed, according to the study conducted by Saafi et al. the scavenging activity of some varieties of dates such as “Khouet Kenta” dates was about 1.96, “Deglet Nour” was 1.53, “Kentichi” was 1.68 and the “Allig” variety was from the lowest level of about 0.72 [58]. Therefore, the Tunisian dates present a higher antiradical activity than the Algerian dates, however, the Saudi by-product dates according to this study reveal a better DPPH scavenging activity.

#### 3.7.3. Ferric Reducing Antioxidant Potential

The ability of the ferric Fe(III)-TPTZ complex to be reduced to the ferrous Fe(II)-TPTZ form in the presence of an antioxidant is preponderant in the evaluation of the ferric reducing antioxidant power and this is manifested by a color change in typical blue. The results in Table 6 showed a high FRAP value of antioxidant activity in the case of our produced date vinegar (1.89 μmol AEAC/mL) compared to the other commercial products (*p* < 0.05). The potent ability of date vinegar to scavenge free radicals (FRAP and DPPH) can be assigned to the presence of a wide range of phenolic compounds such as gallic, ferulic, caffeic, and sinapic acids, procyanidins, and flavonoids [59,60]. Furthermore, the existence of various oligo-compounds as water-soluble antioxidants, and vitamin C is also behind this high antioxidant potential.

#### 3.7.4. Total Carotenoid Content

Carotenoids are colored pigments produced naturally in plants, algae, different bacteria, and fungi. They exhibit a defensive effect on cancer, cardiovascular diseases, and other chronic diseases. The chemical composition of carotenoids is mainly based on a C40 tetraterpenoid structure with a chromophore group responsible for its characteristic color [61]. Results of the β-carotene bleaching assay are shown in Table 6. The registered outcomes indicated a higher antioxidant capacity of the produced date vinegar compared to the commercial vinegars (*p* < 0.05). This recorded antioxidant activity is higher than extracted vinegar from other date varieties such as “Khasab” and “Fard” with total carotenoid contents of 1.31 and 1.39, mg/100 g, respectively [59]. As compared to other extracted or commercially available vinegars, “Khales” date vinegar is a competitive product with excellent antioxidant activity.

## 4. Conclusions

The current study aimed to extract new natural products from a by-product “Khalas” dates as a low-cost and abounded date variety in the Qassim region of Saudi Arabia. The first product was bioethanol as a renewable and alternative biofuel or medical alcohol. Optimization of the production process has led to high purity and valuable quality of bioethanol. The second was vinegar produced with a two-step fermentation process. After optimizing the different production conditions and parameters, the antioxidant activity was evaluated in order to provide more information on its health benefits. Vinegar produced from “Khalas” dates showed significantly (*p* < 0.05) higher phenolic (5.81 mg GAE/mL) and carotenoids values (3.87 mg/100 mL) and better antioxidant performance as compared to some kinds of vinegar extracted from other date varieties and other commercially available vinegars. Therefore, the produced vinegar could be an effective food product with high content of phytochemicals and efficient antioxidant activity. We conclude that the “Khalas” date palm fruit represents a valuable natural source of bioethanol and vinegar with potent antioxidant activity able to prevent many diseases and could potentially be a beneficial additive in food and nutraceutical formulations.

### Future Perspective and Economic Assessment

**Figure 8:**
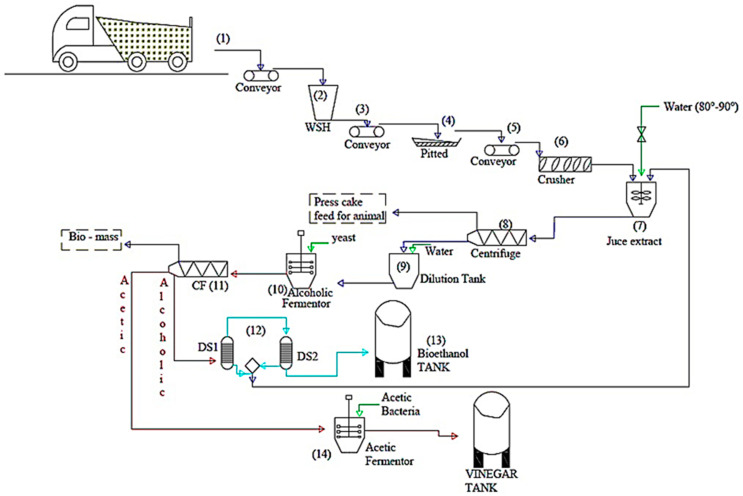
Detailed flow diagram of bioethanol and vinegar production process from waste dates.

The biological production of bioethanol by fermentation of organic materials rich in sugars such as date palm is a possible route. It presents a promising technology thanks to these environmental advantages, its low cost, and its low energy demand. Also, it can have an economic advantage for industrial applications while reducing the problem of the accumulation of organic waste. Accordingly, the research results obtained in this work encourage further research and development in this eco-friendly and sustainable energy. Hence, scientific discoveries in the laboratory could have a profound impact on the economics and sustainability of biofuels and bio-products when implemented at full scale. In fact, the enormous amount of dates lost which is estimated at 80,000 tons each year in Saudi Arabia, is perhaps considered an alternative source of biofuels and energy. Thus, for the industrialization of this fermentation process, a proposed process for ethanol and vinegar production from date fruits was designed, as shown in Figure 8 the process comprises three blocks and five main steps.

The determination of material balances, energies, investment costs, and maintenance is the goal of the next publication.

Bloc A: Pre-treatment; (1) up to (9)

(1) Pre-treatment: pitting dates and shredding raw materials to reduce the size.

(2) Juice extraction using hydrothermal treatment technology; the solid residues (press cake) after juice separation will be considered for animal feed. Afterward, the extracted juice will be clarified and then diluted to an adequate sugar yield concentration.

Bloc B: Bioethanol production; (10) up to (13)

(3) Fermentation of liquid date juice using *Saccharomyces cerevisiae* yeast;

(4) Distillation of the fermentation broth leading to ethanol

Bloc C: Vinegar production; (14)

(5) Acetic fermentation to produce vinegar

## Figures and Tables

**Table 1:** Different synthesized samples.

Code	B10	B15	B25	B50	V15
Samples	Bioethanol	Bioethanol	Bioethanol	Bioethanol	Vinegar
Initial TSS (°Brix)	17 ± 0.19	17 ± 0.19	17 ± 0.2	17 ± 0.2	17 ± 0.2
Yeast concentration (g/L)	10 ± 0.1	15 ± 0.1	25 ± 0.1	50 ± 0.1	15 ± 0.1

**Table 2:** Influence of yeast concentration on bioethanol production.

Code	B10	B15	B25	B50
Yeasts (g/L)	10	15	25	50
Ethanol yield concentration (g/L)	43.45 ± 1.09	60 ± 1.08	58.7 ± 1.02	43 ± 1.02

**Table 3:** Various conditions for ethanol production from dates and comparison with previously published studies.

	Ethanol Production Conditions						
Sugar Concentration (g·L^−1^)	Time (h)	Temp (°C)	pH	Yeast Strains	Shaker (rpm)	Ethanol Concentration (g·L^−1^)	References
162.2	72	30	5	*S. cerevisiae*	200	60 ± 1.08	This work
200	72	30	6	*S. cerevisiae*	150	50	[33]
174	72	28	6	*S. cerevisiae*	382	63	[10]
45	96	30	4	*H. guilliermondii*	150	11	[9]
131.4	48	30	Un-controlled	*S. cerevisiae*	120	48.9	[38]

**Table 4:** Results of physico-chemical analyzes of date vinegar.

Samples	Source	pH	°Brix	Electrical Conductivity (μS/cm)	Ethanol (°)	Acidity (%)
Date vinegar	Saudi Arabia	3.6 ± 0.1	5 ± 0.11	5650 ± 0.2	~0.0 ± 0.05	4 ± 0.1

**Table 5:** 1H NMR chemical shifts and characteristics signals of compounds in vinegar samples.

Compound	Group	δ (ppm)	Multiplicity ^a^	J (Hz)	Hydrogen No.
Acetoin	C4H_3_	1.36	d	7.15	3
Lactic acid	C3H_3_	1.42	d	6.84	3
Acetic acid	C2H_3_	2.1	s	-	3
Succinic acid	C2H_2_ + C3H_2_	2.68	s	-	4
Glucose & Fructose	3CH	3.6	m	-	1
		3.7	m	-	1
Fructose	α(C3H + C5H) + βC5H	4.12	m	-	3
Tartaric acid	C2H + C3H	4.44	s	-	2

^a^ Multiplicity; s, singlet; d, doublet; m, multiple.

**Table 6:** The total phenolic contents, antioxidants, and total carotenoid content of produced date vinegar and comparison with commercial vinegar and other tested date varieties.

Vinegar Sample	TPC (mg GAE/mL)	DPPH (mg TE/mL)	FRAP (μmol AEAC/mL)	TCC (mg/100 mL)
Commercial vinegar	1.12 ± 0.11 ^a^	0.62 ± 0.05 ^b^	0.58 ± 0.08 ^a^	0.88 ± 0.04 ^a^
Deglet Nour (Tunisia)	2.3 ± 0.15 ^c^	1.18 ± 0.09 ^c^	1.62 ± 0.12 ^b^	3.43 ± 0.11 ^b^
Black dates (china)	1.67 ± 0.13 ^b^	0.99 ± 0.02 ^a^	1.27 ± 0.15 ^c^	3.12 ± 0.15 ^c^
Khalas dates (current study)	5.81 ± 0.14 ^bc^	2.01 ± 0.03 ^a^	1.89 ± 0.11 ^b^	3.87 ± 0.12 ^b^

Notes. TPC: total phenolic content; DPPH: 2, 2-diphenyl-1-picrylhydrazyl; FRAP: ferric reducing antioxidant power assay; TCC: total carotenoid contents. Data presented as mean ± SD. Mean values followed by different superscripted alphabets in a column are significantly different at *p* < 0.05.

## Data Availability

Data is contained within the article.
